# Assessment of a training programme for the prevention of ventilator-associated pneumonia

**DOI:** 10.1111/j.1478-5153.2012.00526.x

**Published:** 2012-08-02

**Authors:** M Rosa Jam Gatell, Montserrat Santé Roig, Óscar Hernández Vian, Esther Carrillo Santín, Concepción Turégano Duaso, Inmaculada Fernández Moreno, Jordi Vallés Daunis

**Affiliations:** Critical Care Centre, Hospital de SabadellSabadell, Spain; Critical Care Centre, Hospital de SabadellSabadell, Spain; Primary Care CentreSabadell, Spain; Critical Care Centre, Hospital de SabadellSabadell, Spain; Critical Care Centre, Hospital de SabadellSabadell, Spain; Department of Nosocomial Infection Surveillance and Prevention, Hospital de SabadellSabadell, Spain; Critical Care Centre, Hospital de SabadellSabadell, Spain

**Keywords:** Education, Evidence-based nursing, Intensive care, Observation, Prevention, Ventilator-associated pneumonia

## Abstract

**Background:**

Ventilator-associated pneumonia (VAP) is the most frequent nosocomial infection in intensive care units (ICUs). Most published studies have analysed nurses' theoretical knowledge about a specific procedure; however, the transfer of this knowledge to the practice has received little attention.

**Aim:**

To assess the impact of training session on nurses' knowledge regarding VAP, compliance with VAP preventive measures, VAP incidence and determining whether nursing workload affects compliance.

**Method:**

A prospective, quasiexperimental, pre- and post-study of the nursing team in a 16-bed medical/surgical ICU. *Pre-intervention phase:* a questionnaire to assess nurses' knowledge of VAP prevention measures, direct observation and review of clinical records to assess compliance. *Intervention phase:* eight training sessions for nurses. The post-intervention phase mirrored the pre-intervention phase.

**Findings:**

Nurses answered more questions correctly on the post-intervention questionnaire than on the pre-intervention (17·87 ± 2·69 versus 15·91 ± 2·68, *p* = 0·002). Compliance with the following measures was better during the post-intervention period (*p* = 0·001): use of the smallest possible nasogastric tube, controlled aspiration of subglottic secretions and endotracheal tube cuff pressure, use of oral chlorhexidine and recording the endotracheal tube fixation number. VAP incidence remained unchanged throughout the study. However, a trend towards lower incidence of late (>4 days after intubation) VAP was observed (4·6 versus 3·1 episodes/1000 ventilation days, *p* = 0·37).

**Conclusion:**

The programme improved both knowledge of and compliance with VAP preventive measures, although improved knowledge did not always result in improved compliance.

## INTRODUCTION

Ventilator-associated pneumonia (VAP) is the most frequent nosocomial infection in intensive care units (ICUs; Bregeon *et al.*, 1997; National Nosocomial Infections Surveillance, 2004). VAP is associated with high mortality, longer hospital stays and longer mechanical ventilation (MV), likewise increased costs (Bregeon *et al.*, 1997; Cook, 2000; Bercault and Boulain, 2001; Warren *et al.*, 2003). Risk factors associated with VAP development were grouped as follows: (1) intrinsic factors (individual variable of age, co-morbidity, disease severity, etc.) and (2) extrinsic factors (potential hospital environment risks, prior use of antibiotics, tracheal intubations, etc.; Cook *et al.*, 1998). VAP incidence varies according to risk factors and preventive measures used in ICUs (Chastre and Fagon, 2002; Niederman *et al.*, 2005). Numerous researchers have analysed different drug and non-drug-based strategies to prevent VAP. Likewise, American (Tablan *et al.*, 2004; American Thoracic Society, 2005) and European scientific societies have published evidence-based guidelines for VAP prevention (Torres and Carlet, 2001). Nursing staff play an important role in applying non-drug-based preventive measures directly related to the care they provide; however, adherence to recommendations varies widely (Ibrahim *et al.*, 2001; Rello *et al.*, 2002; Soo Hoo *et al.*, 2005). Ricart *et al.* (2003) reported 22·3% overall non-adherence to these guidelines among nurses attending a critical care congress. Failure to comply has been ascribed to nurses' scientific knowledge (Zack *et al.*, 2002), i.e. routine-based as opposed to evidence-based care (Thompson, 2000; Day *et al.*, 2002; Montial *et al.*, 2005; Williams *et al.*, 2008), resistance to change likewise reluctance to apply some preventive strategies (arguing patient discomfort or potential adverse events; Rello *et al.*, 2002; Ricart *et al.*, 2003) and work overload (Robert *et al.*, 2000; Ricart *et al.*, 2003; Hugonnet *et al.*, 2007).

Many studies have analysed nurses' theoretical knowledge regarding specific procedures (Fulbrook, 2003; Blot *et al.*, 2007). However, the application of this knowledge to practice has received little attention (Chang *et al.*, 2002). Moreover, the instruments used in these studies (e.g. questionnaires) were mainly limited or not entirely appropriate (Cormack and Benton, 1996). Questionnaires may not be reliable measure of compliance as answers may not reflect performance. Thus, it is important to observe nursing practices *in situ*. Few studies have used direct observation to study how nurses apply non-drug-based VAP preventive measures (Day *et al.*, 2002; Montial *et al.*, 2005; Williams *et al.*, 2008). In their study on bed headboard positioning in critical patients, Montial *et al.* (2005) observed that only 25% of nurses placed the headboard according to guidelines (>30°).

The Disease Control Centres (DCCs) consider training a key strategy in reducing VAP incidence and care costs (Thompson, 2000; Zack *et al.*, 2002; Hilary *et al.*, 2004).

The main aim of this study was to assess a training programme to improve nurses' compliance with VAP preventive measures. The secondary objectives were to (a) determine programme impact on nurses' theoretical knowledge of and compliance with the measures, (b) analyse the relationship between workload and compliance and (c) measure programme impact on VAP incidence.

## MATERIAL AND METHODS

A prospective, quasi-experimental, pre- and post-study was carried out in a 16-bed medical-surgical ICU with a mean patient/nurse ratio of 1:2·28 from January 2008 to May 2009. All the ICU nursing staff took part in the study (*n* = 58), excluding the research team. To ensure intervention quality, nursing staff rotation was limited to 15% during the study period.

The study was approved by the hospital ethics committee and participation was voluntary. The variables studied were as follows:

- *Nurses' theoretical knowledge:* An *ad hoc* questionnaire of 22 multiple choice questions was designed following 14 non-drug preventive measures related to nursing care selected from the European Task Force (Torres and Carlet, 2001) and Centers for Disease Control and Prevention (CDC) (Tablan *et al.*, 2004) guidelines. These were grouped into five categories (Table 1). To prevent bias, nurses were asked not to comment on their answers until the last questionnaire had been collected.
- *Nurses' actual performance:* Nurses' adherence to the recommendations was assessed by direct, non-participatory observation and reviewing clinical records. Nurses were aware of the observation, although not the aspects being assessed. Compliance with the 14 measures was assessed and observations made were recorded on a database. To ensure reliability and validity, initial observations were made simultaneously by all the researchers. Consequently, aspiration of endotracheal secretions and headboard positioning were directly observed, comparing the actual inclination with a 45° template. A checklist from the clinical charts was completed after each nursing shift to determine compliance with the other preventive measures.
- *VAP episodes:* number of episodes per 1000 days of MV.
- Workload was measured using the Nine Equivalents of Nursing Manpower Use Score (NEMS), i.e. a questionnaire created to calculate the workload of nursing staff from observations made in 89 ICUs in 12 European countries. The inter-observer interclass correlation was 0·92 (Miranda *et al.*, 1997).
- *Patients' characteristics:* age, Acute Physiology and Chronic Health Evaluation II, Sequential Organ Failure Assessment and days of MV.

**Table 1:** VAP non-drug preventive measures

Preventive measures grouped by categories
1. Procedure for the aspiration of endotracheal secretions
Hand washing before
Hand washing after
Using a sterile catheter
Aseptic manipulation
Changing the catheter for consecutive aspirations
2. Control/reduction in gastric reflux
Smallest possible calibre nasogastric tube
Headboard angle 30°–45°
Controlling gastric retention
3. Preventing microaspiration of subglottic secretions
Controlling the aspiration of subglottic secretions
Emptying subglottic secretions
Controlling the patency of subglottic drainage
Endotracheal tube cuff pressure between 22 and 28 mm Hg
4. Oropharyngeal hygiene
Oral hygiene with 0·12% chlorhexidine
5. Controlling the external fixation of the endotracheal tube
Recording the fixation number

The study was divided into three phases:

– *Pre-intervention phase:* The following aspects were assessed: (a) nurses' basic knowledge using the questionnaire; (b) aspiration of endotracheal secretions during 180 min of non-participatory observation consisting of a systematic sample of 77 observations randomized by day of the week, shift and patient's room; (c) headboard positioning, assessed daily in all three shifts through direct observation and (d) the eight remaining preventive measures on patients' clinical charts, daily in all three shifts.
– *Intervention phase:* The research team designed and held a series of 60-min training sessions covering VAP definition, problem epidemiology and scope, risk factors, aetiology, risk reducing methods and endotracheal secretion aspiration procedure. The session was held eight times to ensure maximum attendance of the three nursing shifts. The session included theoretical training and practical exercises to identify possible errors. In addition, informative posters were displayed in the ICU, a consultation protocol was drawn up and each nurse received a leaflet summarizing the guidelines.
– *Post-intervention phase:* Nurses were assessed identically to the pre-intervention phase.

### Data analysis

Results are presented as mean and standard deviations for quantitative variables, likewise as frequencies and percentages for qualitative variables. The results of the pre- and post-intervention phases were compared using Student's *t*-test for quantitative variables and Pearson's chi-square or Fisher's exact test, as appropriate, for qualitative variables. Statistical significance was set at *p* < 0·05. All analyses were carried out using Statistical Package for the Social Sciences (SPSS) for Windows version 15·0.

## FINDINGS

### Impact on knowledge

The questionnaire was completed by 48 (82·7%) professionals in the pre-intervention phase and 31 (64·5%) in the post-intervention phase.

The mean overall number of correct responses in the post-intervention questionnaire was higher than that in the pre-intervention (17·87 ± 2·69 versus 15·91 ± 2·68; *p* = 0·002). Knowledge about how to prevent microaspiration of subglottic secretions and oropharyngeal hygiene improved significantly (Table 2).

**Table 2:** Nurses' knowledge on VAP non-drug preventive measures: pre- and post-intervention phases

Preventive measures grouped in categories	Scientific knowledge		

	Preinterv. (*n* = 48; %)	Postinterv. (*n* = 31; %)	*p* value
1. Procedure for aspirating endotracheal secretions			
Hand washing before	89·6	100	0·063
Hand washing after	89·6	100	0·063
Using a sterile catheter	75	83·9	0·349
Aseptic manipulation	75	83·9	0·349
Changing the catheter for consecutive aspirations	75	83·9	0·349
2. Control/reduction in gastric reflux			
Smallest possible calibre nasogastric tube	54·2	71	0·135
Headboard angle 30°–45°	97·9	96·7	0·133
Controlling gastric retention	81·3	77·4	0·679
3. Preventing microaspiration of subglottic secretions			
Controlling the aspiration of subglottic secretions	54·2	77·4	0·036
Clearing subglottic secretions	54·2	77·4	0·036
Controlling the patency of subglottic drainage	54·2	77·4	0·036
Endotracheal tube cuff pressure between 22 and 28 mm Hg	91·7	87·1	0·551
4. Oropharyngeal hygiene			
Oral hygiene with 0·12% chlorhexidine	12·5	93·5	0·001
5. Controlling the external fixation of the endotracheal tube			
Recording the fixation number	81·30	87·10	0·494

### Impact on actual performance

For each phase of the study, a chart was drawn up indicating the day, shift and patient. These two charts included 77 randomly selected observations to determine nurses' adherence to guidelines during aspiration of endotracheal secretions. This procedure was observed 67 times in the pre-intervention phase and 111 times in the post-intervention phase. The difference in the number of observations may be due to the procedure not being performed or being performed more than once during the observation period.

Headboard positioning and the other eight preventive measures were checked at the same time on 1145 occasions in the pre-intervention phase and on 1147 occasions in the post-intervention phase (Table 3). The discrepancy in number of observations is due to illegible handwriting on the charts.

**Table 3:** Nurses' compliance with VAP non-drug preventive measures pre- and post-intervention phases

Preventive measures grouped into categories	Practical competence		

	Preinterv. (*n* = 67; %)	Postinterv. (*n* = 111; %)	*p* value
1. Procedure for the aspiration of endotracheal secretions			
Hand washing before	5·7	9·8	0·427
Hand washing after	31·9	34·1	0·807
Using a sterile catheter	99	97·6	0·699
Aseptic manipulation	97	95·1	0·581
Changing the catheter for consecutive aspirations	93	100·0	0·502
	Preinterv. (*n* = 1145; %)	Postinterv. (*n* = 1147; %)	*p* value
2. Control/reduction in gastric reflux			
Smallest possible nasogastric tube	58·3	72·0	0·001
Headboard angle 30°–45°	57·9	82·6	0·001
Controlling gastric retention	97	97·2	0·546
3. Preventing the microaspiration of subglottic secretions			
Controlling the aspiration of subglottic secretions	32·5	68·7	0·001
Clearing subglottic secretions	88·6	90·3	0·192
Controlling the patency of subglottic drainage	43·6	45·9	0·276
Endotracheal tube cuff pressure between 22 and 28 mm Hg	3·0	88·4	0·001
4. Oropharyngeal hygiene			
Oral hygiene with 0·12% chlorhexidine	6·1	96·3	0·001
5. Controlling the external fixation of the endotracheal tube			
Recording the fixation number	13·50	52·70	0·001

We found significant improvements in compliance with use of the narrowest nasogastric tube (*p* = 0·001), headboard positioning (*p* = 0·001), controlled aspiration of subglottic secretions (*p* = 0·001), correct endotracheal cuff pressure (*p* = 0·001), oral hygiene with chlorhexidine (*p* = 0·001) and recording the endotracheal tube fixation (*p* = 0·001).

No significant differences were observed in nurses' scientific knowledge of or compliance with measures in secretion aspiration. Sterile catheters and aseptic manipulation were used in >95%. Interestingly, in the pre-intervention phase, 89·6% of respondents knew the importance of hand washing pre- and post-secretion aspiration; however, pre-aspiration compliance was only 9·8% and post-aspiration was 34·1% (Table 3).

### Workload dependent compliance

Worse compliance was observed with recommendations for the narrowest nasogastric tube, correct endotracheal cuff pressure, gastric retention control, oral hygiene and endotracheal tube fixation in patients requiring more care, measured by NEMS (Table 4).

**Table 4:** Relationship between workload (NEMS) and compliance with VAP non-drug preventive measures

Preventive measures grouped into categories	NEMS		

	Non-adherence	Adherence	*p* value
2. Control/reduction in gastric reflux			
Smallest possible calibre nasogastric tube	32·59 (*n* = 577)	31·91 (*n* = 1143)	0·02
Headboard angle 30°–45°	32·43 (*n* = 518)	32 (*n* = 1199)	0·16
Controlling gastric retention	34·52 (*n* = 48)	32·08 (*n* = 1165)	0·03
3. Preventing the microaspiration of subglottic secretions			
Controlling the aspiration of subglottic secretions	34·59 (*n* = 839)	31·69 (*n* = 880)	0·001
Emptying subglottic secretions	32·65 (*n* = 176)	32·05 (*n* = 1519)	0·172
Controlling the patency of subglottic drainage	34·51 (*n* = 917)	31·64 (*n* = 783)	0·001
Endotracheal tube cuff pressure between 22 and 28 mm Hg	32·66 (*n* = 999)	31·37 (*n* = 710)	0·001
4. Oropharyngeal hygiene			
Oral hygiene with 0·12% chlorhexidine	34·01 (*n* = 76)	31·99 (*n* = 1510)	0·002
5. Controlling the external fixation of the endotracheal tube			
Recording the fixation number	32·46 (*n* = 1211)	31·37 (*n* = 498)	0·001

### Impact on VAP incidence

Overall VAP incidence remained stable (9·9 versus 9·3 episodes/1000 days under MV) throughout the study. However, there was a trend towards lower incidence (>4 days after intubation) VAP (4·6 versus 3·1 episodes/1000 ventilation days, *p* = 0·36).

## DISCUSSION

### Impact on knowledge and compliance

In general terms, the knowledge nurses have acquired regarding VAP preventive measures has significantly increased post-intervention. In particular, the results obtained show that this educational intervention improved ICU nurses' scientific knowledge about measures related to microaspiration of subglottic secretions and oral hygiene, corroborating reports (Zack *et al.*, 2002; Kelleher and Andrews, 2008) that pre-intervention praxis is based more on routine than on scientific evidence. In addition, nurses' compliance with controlling and reducing gastric reflux, preventing microaspiration, oral hygiene and endotracheal tube fixation improved.

As observed in other studies, although oral hygiene procedures were performed more than once a day before the training programme, most nurses did not use chlorhexidine solution (DeRiso *et al.*, 1996; Tablan *et al.*, 2004; Koeman *et al.*, 2006). Cason *et al.* (2007) also found that only 26% of nurses used chlorhexidine. Compliance with recommendations for oropharyngeal hygiene improved after the training programme.

Compliance with both measures related to gastric reflux control (headboard positioning and nasogastric tube diameter) improved. Headboard angle after the intervention was >30° in 82·6% of observations. This percentage is higher than that reported by other authors. Curtis *et al.* (2006), who also compared headboard angle using a template, found the angle was >28° in only 23% of observations. Williams *et al.* (2008) obtained 72% adherence after incorporating a device to demonstrate headboard angle. Lyerla *et al.* (2010), after a training session, found correct headboard positioning in 67% of observations. Our data corroborate previous findings (McMullin *et al.*, 2002; Montial *et al.*, 2005) that nurses tend to overestimate the headboard angle.

Interestingly, nurses knew some VAP preventive measures yet did not apply them (Ricart *et al.*, 2003). In particular, the importance of hand washing pre- and post-secretion aspiration was well known in the pre-intervention phase, as might be expected for a preventive measure with IA evidence (Tablan *et al.*, 2004). In the post-intervention period, a slight improvement was observed. However, adherence remained insufficient considering the rate of morbimortality associated with nosocomial infections in critical patients. Several publications have reported the low adherence to this crucial guideline (Day *et al.*, 2002; Boyce and Pittet, 2003; Creedon, 2005; Williams *et al.*, 2008). Recently, Kelleher and Andrews (2008) have reported 31% adherence to hand washing before aspirating endotracheal secretions. As suggested elsewhere (Boyce and Pittet, 2003), low adherence to this guideline in this study might be due to heavy workload. Cho *et al.* (2003) concluded that a heavy nursing workload contributes to the failure to wash hands and isolate patients with multiresistant organisms. Following international campaigns for patient safety (Pittet *et al.*, 2006), we have introduced more efficient alcohol-based solutions (Pittet *et al.*, 2000; Girou *et al.*, 2002).

Compliance with other recommendations for aspirating endotracheal secretions was better than that referred by other authors: sterile catheters were used in 98% (compared with 59% reported by Kelleher and Andrews, 2008), and as with other studies (Ania *et al.*, 2004; Kelleher and Andrews, 2008), sterile gloves were used in over 95%. The results obtained are not statistically significant, because compliance with these practices was very high before the training intervention and was difficult to improve.

Failure to wash hands when aspirating secretions suggests the persistence of the misconception that wearing gloves makes hand washing unnecessary (Pratt *et al.*, 2001).

### Workload-dependent compliance

Failure to apply guidelines derives from resistance to change, difficulties in accessing literature, lack of resources, costs of the interventions and misinterpretation (Needleman *et al.*, 2002; Rello *et al.*, 2002; Ricart *et al.*, 2003).

The results obtained in this study coincide with the findings of Hugonnet *et al.* (2007), who describe work overload as a reason for not following the guidelines. Thus, compliance with preventive measures decreases as NEMS increases. Six measures were affected: using the narrowest nasogastric tube, gastric reflux control, oral hygiene, aspiration of subglottic secretions, checking the patency of subglottic tubes and correct endotracheal tube fixation. Apparently, when faced with an increased workload, nurses ignore measures they consider less important, such as replacing a nasogastric tube or completing charts thoroughly. Failure to record an action does not mean it was not performed; nursing registers are seldom complete (Alconero *et al.*, 1999).

Moreno and Miranda (1998) propose classifying NEMS results into three groups: group 1 (light workload): NEMS < 21; group 2 (moderate workload): NEMS 21–30 and group 3 (heavy workload): NEMS > 30. All the patients studied were in group 3, i.e. unstable patients requiring a lot of care (Table 4). Despite the small workload differences generated by patients, significant differences were observed in compliance with some preventive measures, probably due to the large number of determinations recorded.

### Impact on VAP

Unlike other studies, which report decreases in VAP incidence after improved compliance with these measures (Zack *et al.*, 2002), we observed no differences in overall VAP incidence pre- and post-intervention (9·9 versus 9·3 episodes/1000 ventilation days). However, late VAP (>4 days of MV) tended to decrease after the intervention (4·6 versus 3·1 episodes/1000 ventilation days). Nurses' preventive measures have a greater impact on late VAP than on early VAP.

The Spanish National Surveillance Study of Nosocomial Infection in the ICU (ENVIN, 2008–2009) showed a decrease in VAP incidence in Spain from 14·95 to 11·44 episodes/1000 days MV in 2008 and 2009, respectively (ENVIN, 2008–2009). Overall VAP incidence was difficult to improve applying only non-drug-related preventive measures because it was already low before the intervention.

### Study limitations

The observational method has inherent limitations. Most importantly, the effect of the ‘observer’ on the ‘observed’. Participants who are aware they are being observed may change their behaviour, introducing the Hawthorne effect bias. Another limitation is that many subjects who completed the first questionnaire did not participate in the second phase because they were no longer working in the ICU. However, we consider this drawback less important, because 64·5% of all possible subjects were enrolled.

Another possible limitation of this study is that the questionnaire used to evaluate nurses' knowledge was not validated. Although there is no standardized way of assessing knowledge, we trust that the results obtained do objectively assess nurses' knowledge on VAP prevention, as the questions included in the questionnaire were based on the guidelines established by the CDC (Tablan *et al.*, 2004) and the European Task Force (Torres and Carlet, 2001).

As this was not a randomized study, other concomitant factors may have influenced the results. Nevertheless, during the study, the antibiotics policy remained constant and changes in the nursing staff were minimal.

As this is a single-centre study, the results obtained cannot be extrapolated to centres with different antibiotic policies or nurse–patient ratios.

One should bear in mind that the results obtained regarding the level of knowledge acquired cannot be exclusively attributed to the educational programme. This fact may be influenced by the memory effect (memory bias), i.e. remembered from completion of the first questionnaire. Nevertheless, this aspect would not explain the statistically significant differences found; therefore, the impact of the teaching programme should be assessed positively. Lastly, due to implementation of the teaching programme, the results cannot be explained as a whole, because the design did not consider periodical series being carried out.

## CONCLUSIONS

The positive results obtained in this study lend support to the CDC's recommendations to reinforce training to improve adherence to VAP preventive strategies. Training activities and evidence-based protocols aimed at ICU nurses, improving the care quality and narrowing the gap between scientific knowledge and actual performance.

The training programme improved ICU nurses' theoretical knowledge and adherence to VAP preventive measures.

The results yielded show that the training programme carried out improved nurses' knowledge and clinical practice regarding VAP preventive strategies. It should be pointed out that information obtained from the two questionnaires clearly shows that nurses' scientific knowledge is not necessarily applied in daily practice, which justifies the need of training strategies to reinforce adherence to preventive measures against VAP.

A new line of research should look into the reasons why ICU nurses do not put into clinical practice the measures they know are important. A change in professional practice will only be possible through in-depth knowledge of the reasons for non-adherence to these guidelines.

## WHAT IS KNOWN ABOUT THIS TOPIC

- VAP, the main nosocomial infection in ICUs, increases duration of hospital stay, cost and patient mortality.
- Nurses are responsible for applying non-pharmacological measures to prevent VAP; however, implementation thereof is not standard.
- The main reasons for the incomplete implementation of non-adherence to preventive measures may be due to heavy workload, a deficient theoretical background and a clinical practice based on established routines as opposed to scientific evidence.

## WHAT THIS PAPER ADDS

- Training programmes improve nurses' adherence to non-pharmacological VAP prevention protocols.
- The direct, passive observation method reveals the current degree of non-pharmacological measures by nurses.
- The study opens a new line for specifically assessing the factors involved in the non-implementation of theoretical knowledge into clinical practice.

## References

[b1] Alconero A, Pérez S, Fernández R, Sola JM (1999). Nursing records on the pain assessment in acute myocardial infarction. Enfermería en Cardiológica.

[b2] American Thoracic Society; Infectious Diseases Society of America (2005). Guidelines for the management of adults with hospital-acquired, ventilator-associated and healthcare-associated pneumonia. American Journal of Respiratory and Critical Care Medicine.

[b3] Ania N, Martinez A, Eseberri M, Margall MA, Asiain MC (2004). Assessment of practice competence and scientific knowledge of ICU nurses in the tracheal suctioning. Enfermería Intensiva.

[b4] Bercault N, Boulain N (2001). Mortality rate attributable to ventilator-associated nosocomial pneumonia in an adult intensive care unit: a prospective case-control study. Critical Care Medicine.

[b5] Blot SI, Labeau S, Vandjick D, Van Aken P, Claes B (2007). Executive Board of the Flemish Society of Critical Care nurses. Evidence-based guidelines for the prevention of ventilator-associated pneumonia: results of a knowledge test among intensive care nurses. Intensive Care Medicine.

[b6] Boyce J, Pittet D (2003). Guideline for hand hygiene in health-care settings. Recommendations of the Healthcare Infection Control Practices Advisory Committee and the HICPAC/SHEA/APIC/IDSA Hand Hygiene Task Force. Morbidity and Mortality Weekly Report.

[b7] Bregeon F, Papazian L, Visconti A, Gregoire R, Thirion X, Gouin F (1997). Relationship of microbiologic diagnostic criteria to morbidity and mortality in patients with ventilator associated pneumonia: comparing adult critical care populations. JAMA.

[b8] Cason CL, Tyner T, Saunders S, Broome L (2007). Centers for Disease Control and Prevention. Nurses' implementation of guidelines for ventilator-associated pneumonia from the Centers for Disease Control and Prevention. American Journal of Critical Care.

[b9] Chang BL, Lee JL, Pearson ML, Kahn KL, Elliott MN, Rubenstein LL (2002). Evaluating quality of nursing care. The gap between theory and practice. Journal of Nursing Administration.

[b10] Chastre J, Fagon JY (2002). Ventilator-associated pneumonia. American Journal of Respiratory and Critical Care Medicine.

[b11] Cho SH, Ketefian S, Barkauskas VH, Smith DG (2003). The effects of nurse staffing on adverse events, morbidity, mortality, and medical costs. Nursing Research.

[b12] Cook D (2000). Ventilator-associated pneumonia. Perspectives in the burden of illness. Intensive Care Medicine.

[b13] Cook DJ, Walter SD, Cook RJ (1998). Incidence of and risk factors for ventilator-associated-pneumonia in critically ill patients. Annals of Internal Medicine.

[b14] Cormack D, Benton D (1996). The Research Process in Nursing.

[b15] Creedon S (2005). Healthcare workers hand decontamination practices: compliance with recommended guidelines. Journal of Advanced Nursing.

[b16] Curtis JR, Cook DJ, Wall RJ (2006). Intensive care unit quality improvement: a “how-to” guide for the interdisciplinary team. Critical Care Medicine.

[b17] Day T, Farnell S, Haynes S, Wainwright S, Wilson-Barnett J (2002). Tracheal suctioning: an exploration of nurses' knowledge and competence in acute and high dependency ward areas. Journal of Advanced Nursing.

[b18] DeRiso AJ, Ladowski JS, Dillon TA, Justice JW, Peterson AC (1996). Chlorhexidine gluconate 0·12% oral rinse reduces the incidence of total nosocomial respiratory infection and nonprophylactic systemic antibiotic use in patients undergoing heart surgery. Chest.

[b19] Fulbrook P (2003). Developing best practice in critical care nursing: knowledge, evidence and practice. Nursing Critical Care.

[b20] Girou E, Loyeau S, Legrand P, Oppein F (2002). Efficacy of handrubbing with alcohol based solution versus standard handwashing with antiseptic soap: randomized clinical trial. British Medical Journal.

[b21] Grupo de trabajo de enfermedades infecciosas de la Sociedad Española de Medicina Intensiva y Unidades Coronarias (ENVIN)

[b22] Hilary M, Babcock MD, Zack JE (2004). An educational intervention to reduce ventilator-associated pneumonia in an integrated health system. Chest.

[b23] Hugonnet S, Chrevolet JC, Pittet D (2007). The effect of workload on infection risk in critically ill patients. Critical Care Medicine.

[b24] Ibrahim EH, Ward S, Sherman G (2001). Experience with a clinical guideline for the treatment of ventilator-associated pneumonia. Critical Care Medicine.

[b25] Kelleher S, Andrews T (2008). An observational study on the open system endotracheal suctioning practices of critical care nurses. Journal of Clinical Nursing.

[b26] Koeman M, Van der Ven AJ, Hak E (2006). Oral decontamination with chlorhexidine reduces the incidence of ventilator-associated pneumonia. American Journal of Respiratory and Critical Care Medicine.

[b27] Lyerla F, LeRouge C, Cooke DA, Turpin D, Wilson L (2010). A nursing clinical decision support system and potential predictors of head-of-bed position for patients receiving mechanical ventilation. American Journal of Critical Care.

[b28] McMullin JP, Cook DJ, Meade MO (2002). Clinical estimation of trunk position among mechanically ventilated patients. Intensive Care Medicine.

[b29] Miranda D, Moreno R, Iapichino G (1997). Nine equivalents of nursing manpower use score (NEMS). Intensive Care of Medicine.

[b30] Montial E, Ruiz de Escudero N, Prieto E (2005). Intuition or exactness? Intuitive positioning of the bed headboard in critical patients. Do we need to measure it?. Enfermería Intensiva.

[b31] Moreno R, Miranda DR (1998). Nursing staff in intensive care in Europe: the mismatch between planning and practice. Chest.

[b32] National Nosocomial Infections Surveillance (NNIS) (2004). System Report, data summary from January 1992 through June 2004, issued October 2004. American Journal of Infection Control.

[b33] Needleman J, Buerhaus P, Mattke S (2002). Nurse-staffing levels and the quality of care in hospitals. New England Journal of Medicine.

[b34] Niederman MS, Craven DE, Bonten MJ (2005). Guidelines for the management of adults with hospital-acquired, ventilator-associated, and healthcare-associated pneumonia. American Journal of Respiratory and Critical Care Medicine.

[b35] Pittet D, Allegranzi B, Storr J, Donaldson L (2006). Clean care is safer care: the global patient safety challenge 2005–2006. International Journal of Infectious Diseases.

[b36] Pittet D, Hugonnet S, Harbarth S (2000). Effectiveness of a hospital-wide programme to improve compliance with hand hygiene. Infection Control Programme. Lancet.

[b37] Pratt RJ, Pellote C, Loveday HP, Robinson N, Smith GW (2001). The epic project: developing national evidence based guidelines for preventing health care associated infections. Phase: guidelines for preventing hospital acquired infections. Journal of Hospital Infection.

[b38] Rello J, Lorente C, Bodí M (2002). Why do physicians not follow evidence-based guidelines for preventing ventilator-associated pneumonia?. Chest.

[b39] Ricart M, Lorente C, Díaz E (2003). Nursing adherence with evidence-based guidelines for preventing ventilator-associated pneumonia. Critical Care Medicine.

[b40] Robert J, Fridking S, Blumberg H (2000). The influence of the composition of nursing staff on primary bloodstream infection rates in a surgical intensive care unit. Infection Control and Hospital Epidemiology.

[b41] Soo Hoo GW, Wen YE, Nguyen TV (2005). Impact of clinical guidelines in the management of severe hospital-acquired pneumonia. Chest.

[b42] Tablan OC, Anderson LJ, Besser R (2004). Guidelines for preventing health-care-associated pneumonia, 2003: recommendations of CDC and the Healthcare Infection Control Practices Advisory Committee. MMWR Recommendations and Reports.

[b43] Thompson L (2000).

[b44] Torres A, Carlet J (2001). Ventilator-associated pneumonia. European Task Force on ventilator-associated pneumonia. European Respiratory Journal.

[b45] Warren DK, Shukla SJ, Olsen MA (2003). Outcome and attributable cost of ventilator-associated pneumonia among intensive care unit patients in a suburban medical center. Critical Care Medicine.

[b46] Williams Z, Chan R, Kelly E (2008). A simple device to increase rates of compliance in maintaining 30-degree head-of-bed elevation in ventilated patients. Critical Care Medicine.

[b47] Zack JE, Garrison T, Trovillon E (2002). Effect of an education program aimed at reducing the occurrence of ventilator-associated pneumonia. Critical Care Medicine.

